# Socioeconomic inequalities in health-related quality of life during the COVID-19 pandemic: a six-country comparison using the EQ-5D-5 L

**DOI:** 10.1007/s11136-026-04285-x

**Published:** 2026-06-08

**Authors:** Joëlle Eijkens, Joshua M. Bonsel, You-Shan Feng, M. F. Bas Janssen, Erica I. Lubetkin, Juanita A. Haagsma

**Affiliations:** 1 Department of Public Health, Erasmus MC University Medical Center Rotterdam, Dr. Molenwaterplein 40, Rotterdam, 3015 GD The Netherlands; 2 Department of Orthopaedics and Sports Medicine, Erasmus MC, Rotterdam, the Netherlands; 3 Institute for Clinical Epidemiology and Applied Biometrics, Medical University of Tübingen, Tübingen, Germany; 4 Section Medical Psychology and Psychotherapy, Department of Psychiatry, Erasmus MC, Rotterdam, the Netherlands; 5 Department of Community Health and Social Medicine, CUNY School of Medicine, New York, NY USA

**Keywords:** Health inequality, Health-related quality of life, EQ-5D-5L, Socio-economic status, COVID-19

## Abstract

**Purpose:**

Despite the growing attention to health inequalities, there is no global consensus on how to measure socio-economic status. This study examined inequalities in health-related quality of life (HRQoL) during the early phase of the COVID-19 pandemic across six countries—China, Italy, the Netherlands, Sweden, the United Kingdom (UK), and the United States (US)—using three SES indicators: education level, income, and work status.

**Methods:**

Between April and June 2020, individuals aged 18–75 years old completed a web-based survey. HRQoL was measured using the EQ-5D-5L Level Sum Score (LSS), where higher scores indicate poorer health. Country-specific differences in LSS across SES groups were assessed using Kruskal-Wallis and Mann-Whitney U tests. Multiple linear regression models, adjusted for age, gender, and chronic conditions, were used to explore associations between SES indicators and HRQoL. No formal correction for multiple testing was applied.

**Results:**

Data from 17,607 respondents were analyzed. In all countries except Italy, individuals with lower education levels reported significantly higher LSS scores. The largest disparity was observed in the UK. In the Netherlands, Sweden, the UK, and the US, lower-income groups also had higher LSS scores, while no such differences were observed in China or Italy. Across all countries, unemployed individuals consistently reported worse HRQoL. Regression analyses confirmed that younger age, chronic conditions, and unemployment were strongly associated with poorer HRQoL.

**Conclusions:**

Substantial SES-related health inequalities in HRQoL were observed during the COVID-19 pandemic, especially in the UK. Work status emerged as a particularly strong and consistent predictor across countries.

**Supplementary Information:**

The online version contains supplementary material available at 10.1007/s11136-026-04285-x.

## Abstract (plain language summary)

This study looked at how people’s quality of life differed based on their social and economic situation during the early months of the COVID-19 pandemic. The people who participated in this study were from six countries: China, Italy, the Netherlands, Sweden, the UK, and the US. We measured people’s socioeconomic status (SES) in three ways: their education level, household income, and whether they were employed or not. More than 17,000 people who were aged 18 to 75 years completed an online survey between April and June 2020. Their quality of life was assessed using a questionnaire that is called the EQ-5D-5L. Based on the answers on the questionnaire, we could give each person a score—higher scores mean poorer quality of life. The results showed that in all countries except Italy, people with lower levels of education tended to report poorer quality of life. The biggest gap between low and high education levels was found in the UK. Income differences also mattered: people with lower incomes in the Netherlands, Sweden, the UK, and the US reported lower quality of life, but this was not the case in China or Italy. Across all six countries, people who were unemployed consistently had lower quality of life than those who were employed.

**Supplementary Information:**

The online version contains supplementary material available at 10.1007/s11136-026-04285-x.

## Introduction

Health inequalities—systematic differences in health outcomes between population groups—remain a major global public health concern. The World Health Organization (WHO) affirms that “the enjoyment of the highest attainable standard of health is one of the fundamental rights of every human being” [1]. Despite this, persistent disparities exist across clinical and public health domains worldwide [2].

A large body of research in health economics has shown a robust socioeconomic gradient in health. Studies have repeatedly found that individuals with higher incomes experience better health, lower mortality, and greater longevity, even after adjusting for other socioeconomic factors [3, 4].

Socioeconomic status (SES) is therefore considered a key structural determinant of health. Economic theories point to multiple pathways underlying these gradients, influencing health outcomes through intermediary factors such as health behaviors, exposure to stress, environmental exposures, and access to care [5, 6]. Individuals with lower SES are more likely to engage in unhealthy behaviors and experience barriers to healthcare, contributing to poorer health outcomes. While SES-related health inequalities are well documented within Europe [7], less is known about their magnitude and nature across different countries.

The COVID-19 pandemic further exposed and potentially exacerbated these disparities by disrupting both structural and intermediary determinants of health. Emerging evidence points to increased unhealthy behaviors among socioeconomically disadvantaged groups [8], widespread income and employment losses, especially in low-income countries [9], and higher rates of infection and mortality among lower-SES populations [10, 11]. Despite the growing attention to health inequalities, there is no global consensus on how to measure SES.

SES is inherently multidimensional and no single measure captures its complexity universally. Indicators such as education, income, work status, and material deprivation vary in relevance across national contexts [12–18]. For example, income is a central SES indicator in high-income countries with stable labor markets and formalized wage structures. It is however more difficult to interpret in economies with large informal sectors or irregular earnings. Similarly, education serves as a strong predictor of health in many regions, but the meaning of educational attainment is shaped by national education systems and regional variation in quality and access to education. Cross-country differences in compulsory schooling years, vocational tracks, and tertiary education complicate direct comparisons of education-based health gradients.

Work status further illustrates these challenges. Employment categories such as “employed” and “unemployed” may obfuscate important contextual differences, such as temporary contracts, or state-supported welfare systems.

Similarly, outcome measures differ. Health-related quality of life (HRQoL), which captures physical, mental, and social well-being, is increasingly used in health inequality research. The EQ-5D-5L, a widely validated tool, offers a standardized measure of HRQoL across populations [20]. While prior studies have demonstrated its sensitivity to education-related differences in selected European countries [19, 21], cross-country comparisons and the role of other SES indicators remain underexplored.

This study addresses these gaps by examining health inequalities in HRQoL, as measured by the EQ-5D-5L, across six countries—China, Italy, the Netherlands, Sweden, the United Kingdom (UK), and the United States (US)—during the first phase of the COVID-19 pandemic. Specifically, it investigates education-related disparities and compares the magnitude of HRQoL differences across alternative SES measures: income and work status.

## Methods

### Study design and study population

This cross-sectional study is a secondary analysis of data from the POPulation health impact of the CORoNavirus disease 2019 (COVID-19) pandemic (POPCORN) study [22]. For this study, we used data that were collected during the first wave of data collected. These first wave data were collected via a web-based survey from April 22 to May 5, 2020, in China, Italy, the Netherlands, the UK, and the US, and from May 26 to June 1 in Sweden. The participants, aged 18 to 75 years, were recruited from the general population by an international market research agency (Dynata). The participants were members of the market research agency’s existing voluntary panels. Therefore, the participants had already provided informed consent to participate in online surveys. Detailed comparisons between each country’s sample demographics and national population statistics have been published previously using the same dataset [22]. Data were collected anonymously.

### Data collection and outcome measures

The survey included questions on demographics, social factors and health related topics. Official translations of the questionnaire instruments were used whenever available. If necessary, the survey was translated into the official language of each country and back into English using translation software. The translations were checked by bilingual native speakers. No missing data were present because the survey design required responses to all items.

The primary outcome was the EQ-5D-5L Level Sum Score (LSS). The EQ-5D-5L is a HRQoL instrument that consists of five dimensions, namely mobility, self-care, usual activities, pain/discomfort and anxiety/depression. Each dimension has five response options representing five levels of severity (1=“no problems”, 2=“slight problems”, 3=“moderate problems”, 4=“severe problems”, 5=“extreme problems”) [23]. A Level Sum Score (LSS) was calculated by summing the scores across all five EQ-5D-5 L dimensions, resulting in a total score ranging from 5 (representing “full health”: 1 + 1+1 + 1+1) to 25 (indicating the “worst possible health state”: 5 + 5+5 + 5+5).

We selected LSS for reasons of comparability and methodological consistency across the six study countries. Unlike EQ-5D-5L index values, which rely on country-specific value sets that differ in e.g. elicitation methods, LSS provides a valuation-free metric based solely on the reported severity levels across the five dimensions. This avoids introducing structural heterogeneity unrelated to actual health differences.

The secondary outcome was the EQ VAS. The EQ VAS is a vertical visual analogue scale that is included in the EQ-5D-5L instrument. It captures a respondent’s overall self-rated health on a scale from 0 to 100, where 100 represents “the best health you can imagine” and 0 represents “the worst health you can imagine”. Respondents are asked to indicate their health today by marking a point on the scale.

### Socio-demographic and health characteristics

The survey also included items on age, gender, education level, income, work status and the presence of chronic health conditions. Chronic health conditions were self-reported and included asthma, chronic bronchitis, severe heart disease, stroke-related impairments, diabetes, chronic rheumatoid arthritis, severe or arthritic back pain, arthrosis-related joint issues (knee or hip), cancer, memory problems due to neurological disorders or aging, depression, anxiety disorders, or other long-term health issues. In this study, respondents were categorized based on the presence of one or more of the listed conditions as having “chronic conditions: yes,” or as having “no chronic conditions” if none were reported.

### SES indicators

SES was assessed using three indicators: education level, income, and work status.

Education level was reported as the highest level of education completed, with country-specific response options. These were recoded according to the International Standard Classification of Education (ISCED-97) into three categories: ‘low’ (ISCED levels 1–2), ‘middle’ (levels 3–4), and ‘high’ (level 5 and above) (see Supplementary Material 1a).

Income referred to annual household income from all sources. Based on national income distributions, respondents were grouped into ‘low’ (bottom 20%), ‘middle’ (middle 60%), and ‘high’ (top 20%) income categories for each country (see Supplementary Material 1b for national thresholds).

Work status was captured using eight response options: “in work: employee,” “in work: self-employed,” “out of work for more than 1 year,” “out of work for less than 1 year,” “looking after others,” “student,” “retired,” and “unable to work.” These were recoded into two categories: ‘Employed’: employee, self-employed, student, or retired; ‘Unemployed’: out of work (less or more than 1 year), looking after others, or unable to work (see Supplementary Material 1c). Our primary rationale for the binary recoding of work status was to distinguish individuals participating in a socially structured daily role (employment, education, or retirement) from those experiencing unemployment or an interruption of regular activity due to ill health or caregiving responsibilities.

### Statistical analysis

Descriptive statistics were used to summarize demographic data. The primary and secondary outcome—EQ-5D-5L LSS and EQ VAS—were reported as medians and interquartile ranges (IQRs) for the total sample and by country. Variations in LSS and EQ VAS across SES groups were interpreted as indicators of health inequalities.

Kruskal-Wallis tests were performed to compare LSS and EQ VAS scores between countries. LSS and EQ VAS scores were then further stratified by SES indicators—education level, income, and work status—and described using medians and IQRs for the total sample and within each country. Differences in LSS and EQ VAS between high and low education levels, and between high and low income groups, were assessed using Mann-Whitney U tests. The same test was used to compare LSS and EQ VAS between employed and unemployed respondents.

To examine associations between demographics, SES indicators, and LSS, multiple linear regression analyses were conducted for the total sample and separately for each country. The variable ‘other’ for gender was excluded from the analysis due to the small sample size (*n* = 34; 0.2%). All models included the SES indicators (education level, income level, and work status) and were adjusted for age, sex, and chronic disease status. These variables were retained in all models regardless of statistical significance to avoid instability associated with stepwise selection procedures and enhance comparability across countries. Next, these analysis were repeated with the EQ VAS as dependent variable to examine associations between demographics, SES indicators, and EQ VAS.

Finally, to assess the relative influence of SES indicators, we evaluated both the presence and magnitude of health inequalities and the strength of associations between SES variables and HRQoL in the regression models.

A p-value of < 0.05 was considered statistically significant. Because the analyses were conducted separately for six countries, three SES indicators and two outcome measures, the number of statistical tests is very large, which may increase the likelihood of Type I error. However, we did not apply formal multiple-testing corrections because our primary aim was not to test isolated hypotheses, but rather to describe and compare patterns of socioeconomic inequalities in HRQoL across countries.

All statistical analyses were carried out using SPSS version 28 for Windows [24].

## Results

### Study population

Of the 19,432 individuals who completed the survey, 1,825 (9.4%) did not report their income and were excluded from the analysis. This subgroup differed significantly from those who did report income in terms of age, gender, chronic disease status, education level, and work status (see Supplementary Material 2). The final analytic sample included 17,607 respondents, with the following country distribution: China (*n* = 3,146; 17.9%), Italy (*n* = 2,866; 16.3%), the Netherlands (*n* = 2,736; 15.5%), Sweden (*n* = 2,839; 16.1%), the UK (*n* = 2,964; 16.8%), and the US (*n* = 3,056; 17.4%). Table 1 shows the demographics of the respondents included in the study. The median age of the study sample ranged from 34.0 in the Chinese sample to 48.0 in the Dutch and Swedish sample, with an overall median of 43.0 (IQR 25.0). Less than half of the respondents were male (47.7%). Chronic conditions were present in 43.5% of the respondents, ranging from 20.4% in the Chinese sample to 56.9% in the Swedish sample. 2769 respondents (15.7%) and 9089 respondents (51.6%) of the total sample had a low and high educational level, respectively. With regards to income, 3774 respondents (21.4%) had a low income and 3165 (18.0%) had a high income in the overall sample. 14,393 respondents (81.7%) of the overall sample were employed against 3214 unemployed respondents (18.3%).

**Table 1:** Socio-demographic and medical characteristics of the study population, total and by country

Age	Median (IQR)	China (*n* = 3146)	Italy (*n* = 2866)	Netherlands (*n* = 2736)	Sweden (*n* = 2839)	UK (*n* = 2964)	US (*n* = 3056)	Total (*n* = 17607)
		34.0 (15.0)	43.0 (22.0)	48.0 (29.0)	48.0 (28.0)	44.0 (25.0)	46.0 (27.0)	43.0 (25.0)
Age range	18–24	478 (15.2%)	225 (7.9%)	265 (9.7%)	253 (8.9%)	275 (9.3%)	334 (10.9%)	1830 (10.4%)
	25–34	1116 (35.5%)	559 (19.5%)	483 (17.7%)	486 (17.1%)	603 (20.3%)	522 (17.1%)	3769 (21.4%)
	35–44	824 (26.2%)	760 (26.5%)	456 (16.7%)	496 (17.5%)	670 (22.6%)	584 (19.1%)	3790 (21.5%)
	45–54	437 (13.9%)	595 (20.8%)	469 (17.1%)	556 (19.6%)	484 (16.3%)	558 (18.3%)	3099 (17.6%)
	55–64	257 (8.2%)	411 (14.3%)	530 (19.4%)	491 (17.3%)	471 (15.9%)	524 (17.1%)	2684 (15.2%)
	65–75	34 (1.1%)	316 (11.0%)	533 (19.5%)	557 (19.6%)	461 (15.6%)	534 (17.5%)	2435 (13.8%)
Gender	Male	1388 (44.1%)	1411 (49.2%)	1389 (50.8%)	1402 (49.4%)	1446 (48.8%)	1354 (44.3%)	8390 (47.7%)
	Female	1750 (55.6%)	1453 (50.7%)	1346 (49.2%)	1430 (50.4%)	1514 (51.1%)	1690 (55.3%)	9183 (52.2%)
	Other	8 (0.3%)	2 (0.1%)	1 (0.0%)	7 (0.2%)	4 (0.1%)	12 (0.4%)	34 (0.2%)
Education level	Low	307 (9.8%)	381 (13.3%)	673 (24.6%)	1143 (40.3%)	68 (2.3%)	197 (6.4%)	2769 (15.7%)
	Middle	963 (30.6%)	1276 (44.5%)	828 (30.3%)	516 (18.2%)	1080 (36.4%)	1086 (35.5%)	5749 (32.7%)
	High	1876 (59.6%)	1209 (42.2%)	1235 (45.1%)	1180 (41.6%)	1816 (61.3%)	1773 (58.0%)	9089 (51.6%)
Income	Low	613 (19.5%)	691 (24.1%)	548 (20.0%)	597 (21.0%)	774 (26.1%)	551 (18.0%)	3774 (21.4%)
	Middle	1800 (57.2%)	1761 (61.4%)	1720 (62.9%)	1789 (63.0%)	1539 (51.9%)	2059 (67.4%)	10,668 (60.6%)
	High	733 (23.3%)	414 (14.4%)	468 (17.1%)	453 (16.0%)	651 (22.0%)	446 (14.6%)	3165 (18.0%)
Work status	Employed	2996 (95.2%)	2235 (78.0%)	2136 (78.1%)	2301 (81.0%)	2405 (81.1%)	2320 (75.9%)	14,393 (81.7%)
	Unemployed	150 (4.8%)	631 (22.0%)	600 (21.9%)	538 (19.0%)	559 (18.9%)	736 (24.1%)	3214 (18.3%)
Chronic conditions	Yes	643 (20.4%)	1129 (39.4%)	1416 (51.8%)	1615 (56.9%)	1302 (43.9%)	1568 (51.3%)	7673 (43.6%)

### Health outcomes

The overall median EQ-5D-5L Level Sum Score (LSS) for the total sample was 6.00 (IQR 3.00). Respondents in China reported the highest HRQoL, with a median LSS of 5.00 (IQR 1.00), followed by those in the Netherlands and the UK, both with a median LSS of 6.00 (IQR 4.00). The highest median LSS scores—indicating poorer HRQoL—were observed in Italy, Sweden, and the US, each with a median of 7.00 (IQRs of 3.00, 4.00, and 4.00, respectively; Table 2). Differences in LSS rank sums across countries were statistically significant (*p* < 0.01).

**Table 2:** Median EQ-5D-5L LSS with IQR per SES indicator, total sample and stratified by country

Median (IQR)		China (n = 3146)	Italy (n = 2866)	Netherlands (n = 2736)	Sweden ( n = 2839)	UK (n = 2964)	US (n = 3056)	Total (n = 17,607)	
	Total	5.00 (1.00)	7.00 (3.00)	6.00 (4.00)	7.00 (4.00)	6.00 (4.00)	7.00 (4.00)	6.00 (3.00)	p < 0.01**
Education	Low	5.00 (1.00)*	6.00 (2.00)	7.00 (5.00)*	7.00 (4.00)*	8.00 (6.00)*	8.00 (7.00)*	7.00 (4.00)*	
	Middle	5.00 (1.00)	7.00 (3.00)	6.00 (4.00)	8.00 (5.00)	7.00 (4.00)	7.00 (4.00)	6.00 (4.00)	
	High	5.00 (1.00)*	7.00 (3.00)	6.00 (3.00)*	7.00 (4.00)*	6.00 (3.00)*	7.00 (4.00)*	6.00 (3.00)*	
Income	Low	5.00 (1.00)	7.00 (2.00)	7.00 (5.00)*	8.00 (5.00)*	7.00 (4.00)*	8.00 (6.00)*	7.00 (4.00)*	
	Middle	5.00 (1.00)	6.00 (3.00)	6.00 (3.00)	7.00 (4.00)	6.00 (3.00)	7.00 (4.00)	6.00 (3.00)	
	High	5.00 (1.00)	7.00 (4.00)	6.00 (4.00)*	6.00 (3.00)*	6.00 (2.00)*	6.00 (3.00)*	6.00 (3.00)*	
Work status	Employed	5.00 (1.00)*	6.00 (3.00)*	6.00 (3.00)*	7.00 (3.00)*	6.00 (3.00)*	6.00 (3.00)*	6.00 (3.00)*	
	Unemployed	6.00 (3.00)*	7.00 (2.00)*	9.00 (6.00)*	9.00 (6.00)*	9.00 (7.00)*	9.00 (7.00)*	8.00 (6.00)*	

*Outcomes in bold differ significantly (p < 0.05) within the SES indicator in the country or total sample

^**^ P-value calculated for the difference in rank sums of LSS across countries

The overall median EQ VAS score for the total sample and by country was 80.0, except for respondents from China who reported a median EQ VAS score of 90.0 (IQR 15.0) (Table 3). Differences in EQ VAS rank sums across countries were also statistically significant (*p* < 0.01).

**Table 3:** Median EQ-5D-5L VAS with IQR per SES indicator, total sample and stratified by country

Median (IQR)		China (n = 3146)	Italy (n = 2866)	Netherlands (n = 2736)	Sweden ( n = 2839)	UK (n = 2964)	US (n = 3056)	Total (n = 17,607)	
	Total	90.00 (15.00)	80.00 (20.00)	80.00 (20.00)	80.00 (30.00)	80.00 (20.00)	80.00 (20.00)	80.00 (20.00)	p < 0.01**
Education	Low	85.00 (10.00)	80.00 (20.00)	80.00 (25.00)	75.00 (30.00)*	75.00 (35.00)*	80.00 (30.00)*	80.00 (25.00)*	
	Middle	90.00 (15.00)	80.00 (20.00)	80.00 (20.00)	75.00 (25.00)	80.00 (30.00)	80.00 (20.00)	80.00 (20.00)	
	High	90.00 (15.00)	80.00 (20.00)	80.00 (20.00)	80.00 (20.00)*	80.00 (20.00)*	85.00 (20.00)*	80.00 (20.00)*	
Income	Low	90.00 (20.00)	80.00 (20.00)	80.00 (25.00)*	70.00 (35.00)*	75.00 (35.00)*	75.00 (35.00)*	80.00 (30.00)*	
	Middle	90.00 (10.00)	80.00 (20.00)	80.00 (20.00)	80.00 (25.00)	80.00 (20.00)	80.00 (20.00)	80.00 (20.00)	
	High	90.00 (15.00)	80.00 (20.00)	80.00 (20.00)*	80.00 (20.00)*	85.00 (15.00)*	85.00 (15.00)*	85.00 (15.00)*	
Work status	Employed	90.00 (15.00)*	80.00 (20.00)*	80.00 (20.00)*	80.00 (20.00)*	80.00 (20.00)*	85.00 (20.00)*	80.00 (20.00)*	
	Unemployed	85.00 (25.00)*	80.00 (25.00)*	70.00 (25.00)*	70.00 (36.25)*	70.00 (30.00)*	75.00 (35.00)*	75.00 (30.00)*	

*Outcomes in bold differ significantly (p < 0.05) within the SES indicator in the country or total sample

^**^ P-value calculated for the difference in rank sums of EQ VAS across countries

### Health outcomes by education level

In the total sample, respondents with a low education level had a median LSS of 7.00 (IQR 4.00) versus a median LSS of 6.00 (IQR 3.00) for respondents with a high education level (*p* < 0.05). Respondents from the Netherlands, the UK, and the US with middle or high education levels had a lower median LSS compared to those with a low education group. For respondents from those countries, there was also a significant difference in the rank sums of the LSS between low and high income groups (*p* < 0.05).The largest difference in median LSS between low and high education groups was observed among respondents from the UK, with a difference of 2 points. For respondents from the Netherlands and the US, this difference was 1 point. Contradictory findings were seen in respondents residing in Italy, the median LSS for respondents with a high education level was 1 point higher than for respondents with a low education level. However, there was no significant difference in rank sums (*p* > 0.05). For respondents residing in China the median LSS was 5.00 (IQR 1.00) regardless of education level, although there was a significant difference in rank sums (*p* < 0.05). Similarly, the median LSS for respondents residing in Sweden was 7.00 (IQR 4.00) for both high and low education levels, with a significant difference in ranks sums (*p* < 0.05) (Table 2).

With regards to the EQ VAS, education showed little overall gradient in the pooled sample. There was only modest country-specific variation for respondents from Sweden UK and US, with a significant difference in the rank sums of the LSS between low and high income groups (*p* < 0.05). (Table 3**)**.

### Health outcomes by income level

In the total sample, respondents with a low income reported a higher median LSS of 7.00 (IQR 4.00), compared to 6.00 (IQR 3.00) among those with a high income (*p* < 0.05). In the Netherlands, Sweden, the UK, and the US, individuals with middle or high income consistently had lower median LSS scores than those with low income, with significant differences in rank sums across income groups (*p* < 0.05). The difference in median LSS between income groups was 1 point in both the Netherlands and the UK, and 2 points in Sweden and the US. In contrast, no differences in median LSS or rank sums were observed in China and Italy by income level (*p* > 0.05) (Table 2).

For the EQ VAS, we observed that respondents with a high income reported higher EQ VAS in the pooled data (85.0; IQR 15.0) compared with respondents with low or middle income (both 80.0; IQR 30 and 20, respectively). In Sweden, the UK, and the US, individuals with middle or high income had consistently higher median EQ VAS scores than those with low income, with significant differences in rank sums across income groups (*p* < 0.05). No differences in median EQ VAS or rank sums were observed in China and Italy by income level (*p* > 0.05) (Table 3).

### Health outcomes by work status

In the total sample, unemployed respondents had a median LSS of 8.00 (IQR 6.00) versus a median LSS of 6.00 (IQR 3.00) for employed respondents (*p* < 0.05). Across all countries, employed respondents had a lower median LSS compared to the median LSS of unemployed respondents. Additionally, the rank sums were significantly different between employed and unemployed respondents in all countries (*p* < 0.05). Employed respondents residing in the Netherlands, UK and US had the largest LSS difference (3 points) compared with the unemployed respondents. In Sweden this difference was 2 points. In Italy and China the difference in median LSS was 1 point (Table 2).

The EQ VAS echoes these findings. Unemployed respondents had lower EQ VAS than employed respondents in the pooled sample as well as in all countries, except for Sweden. Differences in median EQ VAS between work status groups were highest among respondents from the Netherlands, Sweden, UK and US (all 10 points differences).The EQ VAS rank sums were significantly different between employed and unemployed respondents in all countries (*p* < 0.05) (Table 3).

### Association between respondent demographics and SES indicators and the LSS

Table 4 presents the multiple linear regression results for the LSS for the total sample and stratified by country. A positive regression coefficient indicates a higher LSS—reflecting poorer HRQoL—relative to the reference group.

**Table 4:** Multiple linear regression results of participant characteristics, SES indicators and EQ-5D-5 L LSS, total sample and by country

	China			Italy			The Netherlands			Sweden			UK			US			Total		
	b	SE	p-value	b	SE	p-value	b	SE	p-value	b	SE	p-value	b	SE	p-value	b	SE	p-value	b	SE	p-value
Intercept	5.560	0.093	< 0.001	6.408	0.145	< 0.001	6.363	0.188	< 0.001	7.015	0.190	< 0.001	6.521	0.213	< 0.001	7.892	0.209	< 0.001	6.378	0.075	< 0.001
Age	-0.002	0.002	0.406	-0.005	0.003	0.078	-0.016	0.003	< 0.001	-0.015	0.003	< 0.001	-0.011	0.004	0.002	-0.038	0.004	< 0.001	-0.011	0.001	< 0.001
Gender (ref = female)*	-0.055	0.050	0.276	0.012	0.080	0.879	0.011	0.099	0.915	-0.105	0.106	0.320	0.109	0.107	0.308	-0.002	0.115	0.983	0.014	0.039	0.718
Chronic disease (ref = no)	1.848	0.063	< 0.001	2.238	0.081	< 0.001	2.386	0.102	< 0.001	2.627	0.108	< 0.001	2.833	0.109	< 0.001	2.738	0.114	< 0.001	2.693	0.040	< 0.001
Low edu level	-0.092	0.094	0.332	-0.194	0.133	0.145	0.514	0.132	< 0.001	-0.002	0.117	0.987	-0.034	0.360	0.925	0.186	0.244	0.446	0.110	0.059	0.059
Middle edu level	-0.103	0.061	0.091	-0.063	0.087	0.470	0.333	0.115	0.004	0.366	0.148	0.013	0.361	0.116	0.002	0.102	0.125	0.413	0.147	0.045	0.001
Low income level	-0.002	0.071	0.972	0.044	0.099	0.661	0.006	0.130	0.963	0.109	0.134	0.418	-0.213	0.131	0.104	0.461	0.158	0.004	-0.092	0.051	0.070
High income level	0.060	0.064	0.350	0.359	0.117	0.002	0.281	0.170	0.098	-0.659	0.146	< 0.001	-0.568	0.165	0.001	0.083	0.166	0.615	-0.186	0.066	0.005
Employment (ref = employed)	0.881	0.124	< 0.001	0.443	0.103	< 0.001	1.558	0.126	< 0.001	1.794	0.140	< 0.001	2.244	0.144	< 0.001	1.476	0.141	< 0.001	1.630	0.054	< 0.001
R^2^	0.246			0.222			0.270			0.269			0.300			0.250			0.274		

*Only male and female, no ‘other gender’.

Across all countries, the presence of one or more chronic conditions was significantly associated with higher LSS scores. Increasing age was linked to lower LSS (better HRQoL) in all countries and in the total sample, except in China, where no significant association was observed. Gender showed no significant association with LSS in any model.

Among SES indicators, lower education level was significantly associated with higher LSS in the Netherlands and the total sample. In Sweden and the UK, a middle education level—rather than a low level—was positively associated with LSS, suggesting a possible U-shaped relationship between education and HRQoL. No significant education-related associations were found in Italy or the US.

Regarding income, higher income levels were significantly associated with lower LSS (better HRQoL) in Sweden and the UK. Conversely, in the Netherlands and Italy, higher income was unexpectedly associated with higher LSS. In the US, lower income was linked to higher LSS, while no significant income-related associations were observed in China or the total sample.

Unemployment was consistently associated with higher LSS scores across all countries and in the total sample, indicating lower HRQoL among unemployed respondents.

### Association between respondent demographics and SES indicators and the EQ VAS

Table 5 presents the multiple linear regression results for the EQ VAS for the total sample and stratified by country. A negative regression coefficient indicates a lower EQ VAS—reflecting poorer HRQoL—relative to the reference group.

**Table 5:** Multiple linear regression results of participant characteristics, SES indicators and EQ VAS, total sample and by country

	China			Italy			The Netherlands			Sweden			UK			US			Total		
	b	SE	p-value	b	SE	p-value	b	SE	p-value	b	SE	p-value	b	SE	p-value	b	SE	p-value	b	SE	p-value
Intercept	91.72	0.83	< 0.001	87.15	1.02	< 0.001	80.30	1.17	< 0.001	80.10	1.25	< 0.001	79.63	1.24	< 0.001	84.43	1.16	< 0.001	83.90	0.47	< 0.001
Age	-0.14	0.02	< 0.001	-0.09	0.02	< 0.001	0.07	0.02	0.001	0.05	0.02	0.040	0.02	0.02	0.319	0.01	0.02	0.596	-0.02	0.01	0.002
Gender (ref = female)*	1.02	0.45	0.024	-0.36	0.56	0.517	-0.48	0.62	0.432	2.04	0.70	0.004	-0.85	0.62	0.173	0.67	0.64	0.301	0.32	0.25	0.194
Chronic disease (ref = no)	-9.73	0.57	< 0.001	-11.18	0.57	< 0.001	-11.82	0.63	< 0.001	-13.92	0.71	< 0.001	-13.51	0.63	< 0.001	-10.72	0.64	< 0.001	-12.72	0.25	< 0.001
Low edu level	1.45	0.84	0.086	1.65	0.93	0.078	1.40	0.82	0.088	-1.59	0.77	0.039	0.34	2.09	0.871	-0.80	1.36	0.560	-0.74	0.37	0.045
Middle edu level	0.36	0.55	0.505	-0.10	0.61	0.871	0.80	0.71	0.263	-1.16	0.98	0.235	-1.69	0.67	0.012	-0.31	0.70	0.653	-0.48	0.28	0.091
Low income level	-0.08	0.64	0.901	-0.42	0.70	0.550	-0.18	0.81	0.820	-3.35	0.89	< 0.001	3.87	0.76	< 0.001	-4.69	0.88	< 0.001	1.88	0.32	< 0.001
High income level	2.04	0.58	< 0.001	0.002	0.82	0.998	2.35	1.06	0.026	4.03	0.97	< 0.001	6.74	0.95	< 0.001	2.51	0.92	0.007	4.16	0.42	< 0.001
Employment (ref = employed)	-3.44	1.11	0.002	-3.52	0.72	< 0.001	-6.70	0.78	< 0.001	-8.81	0.92	< 0.001	-8.87	0.84	< 0.001	-6.29	0.79	< 0.001	-7.01	0.34	< 0.001
R^2^	0.117			0.140			0.168			0.207			0.231			0.145			0.186		

*Only male and female, no ‘other gender’.

In multivariable linear regression, chronic disease and unemployment were the strongest correlates of poorer EQ VAS across all countries. Presence of a chronic disease was associated with 10–14-point lower EQ VAS. Being unemployed (vs. employed) was likewise associated with 3–9-point lower EQ VAS. Income showed a positive gradient in several settings: high income (vs. low income) was associated with higher EQ VAS in the pooled sample, with the largest effects in the UK and Sweden. Low income showed heterogeneous associations: lower VAS in Sweden and US but higher VAS in the UK; in the pooled model the coefficient was small but positive. Educational level exhibited modest and inconsistent associations: compared with high education, the pooled model indicated slightly lower EQ VAS for low education and a non-significant difference for middle education; within countries, significant differences were limited. Gender was generally not associated with EQ VAS, except for higher EQ VAS scores among males in China and Sweden. Age showed a small overall decrease in VAS per year in the pooled model, with heterogeneity by country (decreases in China and Italy; small increases in the Netherlands and Sweden).

## Discussion

This study aimed to examine health inequalities in HRQoL, as measured by the EQ-5D-5L LSS and EQ VAS, across six countries during the COVID-19 pandemic, focusing on education level as the primary SES indicator. Health inequalities were evident in most countries, with lower HRQoL observed among respondents with lower education levels—except in Italy. The greatest disparities were found in the UK. Structural features of the UK context—such as persistent regional deprivation, longstanding income inequality, and marked differences in employment security—may explain why disparities in HRQoL were especially pronounced.

The secondary aim was to assess whether the magnitude of these inequalities differed by SES measure—education, income, or work status. While education and income showed similar magnitudes of inequality, work status consistently revealed larger disparities.

Our findings align with previous studies using EQ-5D data in China [25], Sweden [26], the Netherlands, and the UK [19]. However, unlike Spronk et al. [19], we did not find educational disparities in Italy. Differences in sample composition or the impact of the COVID-19 pandemic may explain this discrepancy. Consistent with the study by Spronk et al. [19], which was conducted before the COVID-19 pandemic, we found the largest inequalities in the UK and the smallest in Italy.

Regression results showed that lower education was significantly associated with poorer HRQoL, measured with the LSS, in the Netherlands, Sweden and the total sample. In Sweden and the UK, a middle education level was associated with lower HRQoL, suggesting a U-shaped pattern. In China, the relationship was reversed. No associations were found in Italy or the US. Education, as a stable SES measure, may better capture long-term effects on HRQoL compared to income or employment, which can fluctuate over time [27, 28].

While visualizations of marginal effects can be informative for illustrating non-linear associations, the U-shaped education–HRQoL patterns observed in a few countries can already be understood from the regression results presented. In particular, the country-specific coefficients clearly indicate where intermediate education groups exhibit slightly lower HRQoL relative to both lower and higher education categories, and where this pattern is absent. These deviations likely reflect contextual factors such as sample composition, cultural differences in health reporting, or income-related measurement differences rather than systematic reversals of socioeconomic gradients. By interpreting the model estimates directly, we are able to highlight these country-specific nuances without adding additional figures, while maintaining clarity and focus in the presentation of results.

Income-related inequalities were inconsistent across countries. Previous research suggested income may be a stronger SES predictor [29, 30], but our findings challenge that assumption. Household income data, used in this study, may be less reliable due to cultural differences in household composition and incomplete information.

Work status emerged as the most consistent SES indicator associated with HRQoL. Unemployed individuals reported significantly lower HRQoL across all countries. This aligns with existing literature showing a widening gap in health between employed and unemployed individuals [31–33]. Employment positively influences mental and physical health [34], but a “healthy worker effect” may confound these findings: healthier individuals are more likely to be employed [35]. Because poorer health can lead to job loss or labor-market withdrawal, differences in HRQoL between employed and unemployed groups may partly reflect underlying health selection rather than socioeconomic disadvantage alone. This dynamic may therefore exaggerate observed disparities and complicates interpretation of employment-related SES gradients.

In our study we used both the EQ-5D-5L LSS and the EQ VAS as outcome measure. Overall, the regression analyses for both the EQ-5D-5L LSS and the EQ VAS showed remarkably consistent patterns, identifying the same key determinants of lower HRQoL across countries. Chronic disease and unemployment emerged as the strongest and most consistent predictors of poorer health across both measures. Chronic disease was associated with substantially poorer outcomes (higher LSS; lower EQ VAS) in all countries, and the magnitude of effects was very similar across the two instruments. Likewise, unemployment showed large and statistically significant associations with poorer health in both models, with highly comparable effect sizes.

In contrast, education and income level showed more heterogeneous associations, and the two instruments did not always align. The EQ VAS appears more responsive to income- and education-related differences, whereas the LSS yields more conservative, and sometimes inconsistent, SES effects across countries. This indicates that while the EQ-5D-5L LSS and EQ VAS capture similar determinants of HRQoL, they differ in their sensitivity to socioeconomic gradients.

The findings of this study have important implications for policy makers that aim to reduce socioeconomic inequalities in health and improve population HRQoL. First, the consistent SES-related disparities observed across countries, even under the exceptional conditions of the COVID-19 pandemic, highlight the need for governments to prioritize structural policies that address education access, income security, and labor market protections. Investments in social safety nets, progressive income support, and targeted interventions for low SES groups may help mitigate the persistent HRQoL gaps identified here.

### Strengths and Limitations

Key strengths of this study include a large, multi-country sample and use of a validated HRQoL instrument. However, a key limitation of this study is its cross-sectional design, which captures exposures and outcomes at a single point in time. As a result, the temporal ordering of socioeconomic status and health-related quality of life cannot be established, and causal inferences cannot be drawn. Observed associations may reflect reverse causality. For example, poorer health leading to lower income or reduced employment. Longitudinal data would be necessary to clarify the directionality of these relationships. Lack of pre-pandemic data makes it difficult to assess whether inequalities worsened during COVID-19.

Second, missing income data from respondents who chose not to disclose may have introduced bias. Approximately 10% of respondents did not report their household income and were therefore excluded from the income-based analyses. Because income non-response may be systematically related to sociodemographic or health characteristics, this exclusion may introduce bias and potentially distort the magnitude of observed income-related inequalities in HRQoL. Although multiple imputation could, in principle, offer a more robust way of addressing missing income, implementing a unified imputation model was complicated by substantial cross-country differences in income categorization, response patterns, and the cultural sensitivity of income reporting. To maintain comparability across countries, we opted for a complete-case approach but acknowledge that future studies should consider advanced missing data techniques, such as multiple imputation, to enhance the robustness of income-related analyses.

A third limitation concerns the online recruitment strategy, which relied on a market research agency. Although this approach enabled rapid data collection during the early phase of the pandemic, it may have introduced selection bias, particularly in countries like China, where the sample skewed younger and healthier. Individuals who join online survey panels tend to be younger, more digitally literate, and may have higher socioeconomic status compared with the general population. As a result, certain groups—such as older adults, people with limited internet access, or individuals with lower literacy levels—may be underrepresented. This potential sampling bias could affect the generalizability of our findings, particularly with regard to the magnitude of socioeconomic inequalities in HRQoL.

Another limitation concerns the operationalization of SES, in particular work status. To ensure sufficient sample sizes across categories, we collapsed several work status groups (i.e., respondents who were employed, students and retirees), which may have obscured important distinctions between different forms of employment and non-employment. Moreover, differences in how countries conceptualize employment, particularly for students and retirees, may comparability and interpretation.

A further limitation of this study is the use of the EQ-5D-5L LSS rather than utility values. Although the LSS provides a simple and transparent summary of respondents’ health states, it is not directly comparable to utility scores derived from country-specific value sets. Utility values are widely used in health economics for cost-utility analyses, and substituting the LSS limits comparability with studies that employ standard EQ-5D utilities. This reduces the extent to which our findings can be integrated into economic evaluation frameworks and cross-study comparisons in the health economics literature.

Cultural differences in self-reporting EQ-5D-5L and EQ VAS may also have influenced outcomes, warranting further investigation.

A final limitation relates to the COVID-19 pandemic during which the data were collected. The pandemic may have temporarily altered socioeconomic conditions and HRQoL, raising questions about the generalizability of our findings over time. Moreover, our analysis does not fully account for alternative macro-level factors that may contribute to cross-country differences, such as variations in health system resilience, welfare state structures, and national COVID-19 response strategies. These institutional and policy environments likely shaped countries’ capacity to buffer populations from healthcare disruptions, income loss, and wider social consequences, potentially influencing the magnitude of observed SES-related disparities. Nevertheless, the relative socioeconomic gradients we identify align with longstanding evidence on health inequalities, suggesting they reflect deeper structural mechanisms rather than pandemic-specific effects. Still, because absolute HRQoL levels and the extent of inequalities may evolve as countries recover, future studies incorporating post-pandemic data and explicit contextual indicators will be essential to assess the persistence, change, and drivers of these cross-country patterns.

## Conclusions

Health inequalities in HRQoL during the COVID-19 pandemic were evident in China, the Netherlands, Sweden, the UK, and the US, with the UK showing the greatest disparities. Education and income were similarly associated with HRQoL, but work status appeared to be a more powerful predictor. Although the cross-sectional design limits causal interpretation, the consistent SES-related gradients observed across diverse contexts highlight persistent structural. These findings reinforce the need for governments to prioritize policies that reduce socioeconomic vulnerability, such as strengthening income protection, improving employment security, and expanding access to education and social support.

## Supplementary Information

Below is the link to the electronic supplementary material.

Supplementary Material 1

Supplementary Material 2

## Data Availability

Data is available upon reasonable request.
