# Supplementary material for: Socioeconomic inequalities in health-related quality of life during the COVID-19 pandemic: a six-country comparison using the EQ-5D-5 L
Source: Qual Life Res. 2026 Jun 8;35(7):188. doi: 10.1007/s11136-026-04285-x (PMC13246841; doi:10.1007/s11136-026-04285-x)
Supplement: Supplementary file 2 — Supplementary Material 2 [file 11136_2026_4285_MOESM2_ESM.docx]

**Supplementary material**

**Title manuscript: Socioeconomic Inequalities in Health-Related Quality of Life During the COVID-19 Pandemic: A Six-Country Comparison Using the EQ-5D-5L**

**Supplementary material 1a. Categorizing level of education**

The variable level of education was recoded using the International Standard Classification of Education (ISCED-97). Each country was categorized separately to ultimately form 3 categories: low (ISCED 0, 1 and 2), middle (ISCED 3 and 4) and high (ISCED 5 and higher).

*China:*

|  | **ISCED** | **Category** |
| --- | --- | --- |
| Primary school | 1 | Low |
| Junior middle school | 2 | Low |
| Senior high school or vocational | 3 | Middle |
| University or college | 5 | High |
| Postgraduate education completed | 5 | High |
| Doctorate, post-doctorate or equivalent completed | 6 | High |

*Italy*:

|  | **ISCED** | **Category** |
| --- | --- | --- |
| Scuola media inferior | 1 | Low |
| Scuola elementaire | 1 | Low |
| Istituto professionale | 3 | Middle |
| Scuola superior | 3 | Middle |
| Universita | 5 | High |
| Master | 5 | High |
| Dottorato | 6 | High |

*The Netherlands:*

|  | **ISCED** | **Category** |
| --- | --- | --- |
| Lagere school | 1 | Low |
| LBO (LTS, huishoudschool etc.) | 2 | Low |
| MAO (MAVO, 3 jr VWO etc.) | 2 | Low |
| MBO | 3 | Middle |
| HAO | 3 | Middle |
| HBO | 6 | High |
| Wetenschappelijk onderwijs (universiteit) | 6 | High |

*Sweden:*

|  | **ISCED** | **Category** |
| --- | --- | --- |
| Primary education (Year 1-9) completed | 1 | Low |
| Secondary education completed | 2 | Low |
| KY Vocational education or other vocational education | 3 | Middle |
| University – Academic education ground level (BA) | 5 | High |
| University – Academic education advanced level (MA) | 5 | High |
| University – Academic education doctoral level (PhD) | 6 | High |

*United Kingdom:*

|  | **ISCED** | **Category** |
| --- | --- | --- |
| Combined Junior and Infant School/ Infant School | 1 | Low |
| Junior school | 1 | Low |
| Comprehensive school | 3 | Middle |
| Comprehensive School (GCSE)/ Secondary Modern (GCSE)/ Grammar School (GSCE)/ City Technology College (CGSE)/ Sixth Form | 3 | Middle |
| College and Institution of Higher education | 5 | High |
| Open College -College of Technology - Institute/ Teacher Training College | 5 | High |
| University/ Open University | 6 | High |

*United States of America:*

|  | **ISCED** | **Category** |
| --- | --- | --- |
| Incomplete secondary education | 2 | Low |
| Secondary education completed | 3 | Middle |
| Some university or vocational certification | 4 | Middle |
| Vocational or professional certification completed | 5 | High |
| University education complete | 6 | High |
| Postgraduate education completed | 6 | High |
| Doctorate, post-doctorate or equivalent completed | 6 | High |

**Supplementary material 1b. Categorizing income**

*China:*

|  | **Cumulative percent** | **Category** |
| --- | --- | --- |
| < 15.000 CNY | 4.7% | Low |
| 15.000 CNY – 24.999 CNY | 8.4% | Low |
| 25.000 CNY – 34.999 CNY | 10.9% | Low |
| 35.000 CNY – 44.999 CNY | 13.7% | Low |
| 45.000 CNY – 54.999 CNY | 16.7% | Low |
| 55.000 CNY – 64.999 CNY | 19.5% | Middle |
| 65.000 CNY – 74.999 CNY | 23.0% | Middle |
| 75.000 CNY – 84.999 CNY | 27.1% | Middle |
| 85.000 CNY – 94.999 CNY | 32.6% | Middle |
| 95.000 CNY – 99.999 CNY | 41.7% | Middle |
| 100.000 CNY – 149.999 CNY | 62.7% | Middle |
| 150.000 CNY – 199.999 CNY | 76.7% | Middle |
| 200.000 CNY – 249.999 CNY | 88.4% | High |
| > 250.000 CNY | 100.0% | High |

Median annual household income *per capita* in China in 2020: 27,540 CNY (2).

*Italy:*

|  | **Cumulative percent** | **Category** |
| --- | --- | --- |
| < €20.000 | 24.1% | Low |
| €20.000 – €39.999 | 68.6% | Middle |
| €40.000 – €59.999 | 85.6% | Middle |
| €60.000 – €79.999 | 92.8% | High |
| €80.000 – €99.999 | 96.1% | High |
| €100.000 – €149.000 | 97.7% | High |
| €150.000 – €299.999 | 98.6% | High |
| €300.000 – €499.999 | 98.9% | High |
| > €500.000 | 100.0% | High |

Median annual income per household in Italy in 2020: €32,812 (2).

*The Netherlands:*

|  | **Cumulative percent** | **Category** |
| --- | --- | --- |
| < €20.000 | 20.0% | Low |
| €20.000 – €39.999 | 50.4% | Middle |
| €40.000 – €59.999 | 70.6% | Middle |
| €60.000 – €79.999 | 82.9% | Middle |
| €80.000 – €99.999 | 92.1% | High |
| €100.000 – €149.000 | 97.0% | High |
| €150.000 – €299.999 | 98.9% | High |
| €300.000 – €499.999 | 99.4% | High |
| > €500.000 | 100.0% | High |

Median annual income per household in the Netherlands in 2020: €46,800 (3).

*Sweden:*

|  | **Cumulative percent** | **Category** |
| --- | --- | --- |
| < 100.000 kr | 6.8% | Low |
| 100.000 kr – 199.999 kr | 21.0% | Low |
| 200.000 kr – 299.999 kr | 36.7% | Middle |
| 300.000 kr – 399.999 kr | 52.4% | Middle |
| 400.000 kr – 499.999 kr | 66.1% | Middle |
| 500.000 kr – 599.999 kr | 75.9% | Middle |
| 600.000 kr – 699.999 kr | 84.0% | Middle |
| 700.000 kr – 799.999 kr | 90.0% | High |
| 800.000 kr – 899.999 kr | 94.0% | High |
| 900.000 kr – 999.999 kr | 96.1% | High |
| >1.000.000 kr | 100.0% | High |

Median annual income per household in Sweden in 2020: 427.500 kr (4).

*United Kingdom:*

|  | **Cumulative percent** | **Category** |
| --- | --- | --- |
| < £14.000 | 13.0% | Low |
| £14.000 – £20.999 | 26.1% | Low |
| £21.000 – £27.999 | 38.9% | Middle |
| £28.000 – £34.999 | 51.7% | Middle |
| £35.000 – £41.999 | 62.8% | Middle |
| £42.000 – £48.999 | 70.9% | Middle |
| £49.000 – £55.999 | 78.0% | Middle |
| £56.000 – £62.999 | 82.8% | High |
| £63.000 – £69.999 | 87.1% | High |
| £70.000 – £90.999 | 97.0% | High |
| > £91.000 | 100.0% | High |

Median annual income per household in the UK in 2020: £30,500 (5).

*United States of America:*

|  | **Cumulative percent** | **Category** |
| --- | --- | --- |
| < 15.000$ | 9.7% | Low |
| 15.000$ - 24.999$ | 18.0% | Low |
| 25.000$ – 49.999$ | 37.4% | Middle |
| 50.000$ – 74.999$ | 56.5% | Middle |
| 75.000$ – 99.999$ | 70.2% | Middle |
| 100.000$ – 149.000$ | 85.4% | Middle |
| 150.000$ – 199.999$ | 92.2% | High |
| 200.000$ – 249.999$ | 95.2% | High |
| 250.000$ - 499.999$ | 98.3% | High |
| 500.000$ – 999.999$ | 99.2% | High |
| > 1.000.000$ | 100.0% | High |

Median annual income per household in the US in 2020: $67,521 (6).

National Bureau of Statistics of China. Households’ Income and Consumption Expenditure in 2020 [Internet]. Available from: [Households' Income and Consumption Expenditure in 2020 (stats.gov.cn)](https://www.stats.gov.cn/english/PressRelease/202101/t20210119_1812523.html). [Accessed 25th July 2024].

Istituto Nazionale di Statistica. Condizioni di vita e reddito delle famiglie – Anni 2020-2021 [Internet]. Available from: <https://www.istat.it/comunicato-stampa/condizioni-di-vita-e-reddito-delle-famiglie-anni-2020-e-2021>. [Accessed 25th July 2024].

Centraal Bureau voor de Statistiek. Materiële welvaart in Nederland 2022 [Internet]. Available from: [Inkomen van huishoudens - Materiële welvaart in Nederland 2022 | CBS](https://longreads.cbs.nl/materiele-welvaart-in-nederland-2022/inkomen-van-huishoudens/). [Accessed 25th July 2024].

Statistics Sweden. Income and tax statistics [Internet]. Available from: https://www.scb.se/he0110-en. [Accessed 25th July 2024].

Office for National Statistics. Average household income, UK: financial year 2020 [Internet]. Available from: [Average household income, UK - Office for National Statistics (ons.gov.uk)](https://www.ons.gov.uk/peoplepopulationandcommunity/personalandhouseholdfinances/incomeandwealth/bulletins/householddisposableincomeandinequality/financialyear2020). [Accessed 25^th^ July 2024].

Shrider, Emily A., Kollar M, Chen F, Semega J. U.S. Census Bureau, Current Population Reports, P60-273, Income and Poverty in the United States: 2020, U.S. Government Publishing Office, Washington, DC, September 2021.

**Supplementary material 1c. Categorizing income**

**Categorizing work status**

|  | **Category** |
| --- | --- |
| In work: employee | Employed |
| In work: self-employed | Employed |
| Out of work for more than 1 year | Unemployed |
| Out of work for less than 1 year | Unemployed |
| Looking after others (e.g. carer or parent) | Unemployed |
| A student | Employed |
| Retired | Employed |
| Unable to work | Unemployed |

**Supplementary material 2. Demographics participants without reported income**

|  | | Income reported | Income not reported | p-value |
| --- | --- | --- | --- | --- |
| Age (median) |  | 43.0 (14.0) | 47.0 (15.0) | <0.001^■^ |
| Gender | Male | 8390 (47.7%) | 658 (36.1%) | <0.001* |
|  | Female | 9183 (52.2%) | 1157 (63.4%) |  |
|  | Other | 34 (0.2%) | 10 (0.5%) |  |
| Chronic conditions | Yes | 7673 (43.6%) | 694 (38.0%) | <0.001* |
|  | No | 9934 (56.4%) | 1131 (62.0%) |  |
| Education level | Low | 2769 (15.7%) | 473 (25.9%) | <0.001* |
|  | Middle | 5749 (32.7%) | 597 (32.7%) |  |
|  | High | 9089 (51.6%) | 755 (41.4%) |  |
| Work status | Employed | 14393 (81.7%) | 1359 (74.5%) | <0.001* |
|  | Unemployed | 3214 (18.3%) | 466 (25.5%) |  |

^■^ *The p-value was obtained using a Mann-Whitney U Test*
**The p-value was obtained using a chi-squared test*

**Supplementary material 3. Chronic disease status per education level**

## *Chronic condition status*

Supplementary Material 3 presents the proportion of respondents with a chronic disease, stratified by education level and country. The distribution of education levels among those with and without chronic conditions differed significantly (p < 0.001). The highest proportions of chronic disease were observed among respondents with low education levels in the UK (67.6%), the Netherlands (66.3%), the US (59.9%), and Italy (41.2%). In contrast, in China, the highest proportion of chronic disease was found among respondents with a high education level (23.5%). Notably, the prevalence of chronic disease among low-educated respondents in China was lower than that of any education subgroup in any other country. In Sweden, the highest proportion of chronic disease was observed in the middle education group (65.5%).

| *Total* | | | Chronic disease | Healthy | Total | P-value |
| --- | --- | --- | --- | --- | --- | --- |
| Education Level | Low | Count | 1506 | 1263 | 2769 | <0.001* |
|  |  | % within Education Level | 54.4% | 45.6% | 100.0% |  |
|  | Middle | Count | 2520 | 3229 | 5749 |  |
|  |  | % within Education Level | 43.8% | 56.2% | 100.0% |  |
|  | High | Count | 3647 | 5442 | 9089 |  |
|  |  | % within Education Level | 40.1% | 59.9% | 100.0% |  |
| Total | | Count | 7673 | 9934 | 17607 |  |
|  |  | % within Education Level | 43.6% | 56.4% | 100.0% |  |

**The p-value was obtained using a chi-squared test*

| *China* | | | Chronic disease | Healthy | Total | P-value |
| --- | --- | --- | --- | --- | --- | --- |
| Education Level | Low | Count | 61 | 246 | 307 | <0.001 |
|  |  | % within Education Level | 19.9% | 80.1% | 100.0% |  |
|  | Middle | Count | 142 | 821 | 963 |  |
|  |  | % within Education Level | 14.7% | 85.3% | 100.0% |  |
|  | High | Count | 440 | 1436 | 1876 |  |
|  |  | % within Education Level | 23.5% | 76.5% | 100.0% |  |
| Total | | Count | 643 | 2503 | 3146 |  |
|  |  | % within Education Level | 20.4% | 79.6% | 100.0% |  |

**The p-value was obtained using a chi-squared test*

| *Italy* | | | Chronic disease | Healthy | Total | P-value |
| --- | --- | --- | --- | --- | --- | --- |
| Education level | Low | Count | 157 | 224 | 381 | 0.599 |
|  |  | % within Education level | 41,2% | 58,8% | 100,0% |  |
|  | Middle | Count | 507 | 769 | 1276 |  |
|  |  | % within Education level | 39,7% | 60,3% | 100,0% |  |
|  | High | Count | 465 | 744 | 1209 |  |
|  |  | % within Education level | 38,5% | 61,5% | 100,0% |  |
| Total | | Count | 1129 | 1737 | 2866 |  |
|  |  | % within Education level | 39,4% | 60,6% | 100,0% |  |

**The p-value was obtained using a chi-squared test*

| The Netherlands | | | | | Chronic disease | | Healthy | | Total | | P-value | |  |
| --- | --- | --- | --- | --- | --- | --- | --- | --- | --- | --- | --- | --- | --- |
| Education Level | Low | | Count | | 446 | | 227 | | 673 | | <0.001 | |  |
|  |  |  | % within Education Level | | 66,3% | | 33,7% | | 100,0% | |  |  |  |
|  | Middle | | Count | | 428 | | 400 | | 828 | |  |  |  |
|  |  |  | % within Education Level | | 51,7% | | 48,3% | | 100,0% | |  |  |  |
|  | High | | Count | | 542 | | 693 | | 1235 | |  |  |  |
|  |  |  | % within Education Level | | 43,9% | | 56,1% | | 100,0% | |  |  |  |
| Total | | | Count | | 1416 | | 1320 | | 2736 | |  |  |  |
|  |  |  | % within Education Level | | 51,8% | | 48,2% | | 100,0% | |  |  |  |
| **The p-value was obtained using a chi-squared test* | | | | | | | | | | | |  | |
| Sweden | | | | | | Chronic disease | | Healthy | | Total | | P-value | |
| Education level | | Low | | Count | | 678 | | 465 | | 1143 | | <0.001 | |
|  |  |  |  | % within Education level | | 59,3% | | 40,7% | | 100,0% | |  |  |
|  |  | Middle | | Count | | 338 | | 178 | | 516 | |  |  |
|  |  |  |  | % within Education level | | 65,5% | | 34,5% | | 100,0% | |  |  |
|  |  | High | | Count | | 599 | | 581 | | 1180 | |  |  |
|  |  |  |  | % within Education level | | 50,8% | | 49,2% | | 100,0% | |  |  |
| Total | | | | Count | | 1615 | | 1224 | | 2839 | |  |  |
|  |  |  |  | % within Education level | | 56,9% | | 43,1% | | 100,0% | |  |  |

**The p-value was obtained using a chi-squared test*

| UK | | | Chronic disease | Healthy | Total | P-value |
| --- | --- | --- | --- | --- | --- | --- |
| Education level | Low | Count | 46 | 22 | 68 | <0.001 |
|  |  | % within Education level | 67,6% | 32,4% | 100,0% |  |
|  | Middle | Count | 502 | 578 | 1080 |  |
|  |  | % within Education level | 46,5% | 53,5% | 100,0% |  |
|  | High | Count | 754 | 1062 | 1816 |  |
|  |  | % within Education level | 41,5% | 58,5% | 100,0% |  |
| Total | | Count | 1302 | 1662 | 2964 |  |
|  |  | % within Education level | 43,9% | 56,1% | 100,0% |  |

**The p-value was obtained using a chi-squared test*

| US | | | Chronic disease | Healthy | Total | P-value |
| --- | --- | --- | --- | --- | --- | --- |
| Education Level | Low | Count | 118 | 79 | 197 | <0.001 |
|  |  | % within Education Level | 59,9% | 40,1% | 100,0% |  |
|  | Middle | Count | 603 | 483 | 1086 |  |
|  |  | % within Education Level | 55,5% | 44,5% | 100,0% |  |
|  | High | Count | 847 | 926 | 1773 |  |
|  |  | % within Education Level | 47,8% | 52,2% | 100,0% |  |
| Total | | Count | 1568 | 1488 | 3056 |  |
|  |  | % within Education Level | 51,3% | 48,7% | 100,0% |  |

**The p-value was obtained using a chi-squared test*
